# Targeting T cell malignancies using CAR-based immunotherapy: challenges and potential solutions

**DOI:** 10.1186/s13045-019-0801-y

**Published:** 2019-12-29

**Authors:** Lauren C. Fleischer, H. Trent Spencer, Sunil S. Raikar

**Affiliations:** 1 Molecular and Systems Pharmacology Graduate Program, Graduate Division of Biological and Biomedical Sciences, Laney Graduate School, Emory University School of Medicine, Atlanta, GA USA; 2 Cell and Gene Therapy Program, Department of Pediatrics, Aflac Cancer and Blood Disorders Center, Children’s Healthcare of Atlanta and Emory University School of Medicine, Atlanta, GA USA

**Keywords:** CAR, Immunotherapy, T-ALL, T cell lymphoma

## Abstract

Chimeric antigen receptor (CAR) T cell therapy has been successful in treating B cell malignancies in clinical trials; however, fewer studies have evaluated CAR T cell therapy for the treatment of T cell malignancies. There are many challenges in translating this therapy for T cell disease, including fratricide, T cell aplasia, and product contamination. To the best of our knowledge, no tumor-specific antigen has been identified with universal expression on cancerous T cells, hindering CAR T cell therapy for these malignancies. Numerous approaches have been assessed to address each of these challenges, such as (i) disrupting target antigen expression on CAR-modified T cells, (ii) targeting antigens with limited expression on T cells, and (iii) using third party donor cells that are either non-alloreactive or have been genome edited at the T cell receptor α constant (TRAC) locus. In this review, we discuss CAR approaches that have been explored both in preclinical and clinical studies targeting T cell antigens, as well as examine other potential strategies that can be used to successfully translate this therapy for T cell disease.

## Introduction

T cell malignancies encompass a heterogeneous group of diseases, each reflecting a clonal evolution of dysfunctional T cells at various stages of development. T cell acute lymphoblastic leukemia (T-ALL) accounts for 15% and 25% of childhood and adult ALL cases respectively, and is the most common form of T cell cancer seen in children [1, 2]. T-lymphoblastic lymphoma (T-LLy) is a non-Hodgkin lymphoma with similar biology to T-ALL. Adult T cell leukemia/lymphoma (ATLL) is an extremely aggressive form of blood cancer driven by the human T cell lymphocytic virus type 1 (HTLV1) [3–5]. Other rare forms of T cell leukemia include T cell large granular lymphocytic leukemia (T-LGL) and T-prolymphocytic leukemia (T-PLL) [6]. T cell lymphomas are broadly divided into two categories, cutaneous T cell lymphoma (CTCL) and peripheral T cell lymphoma (PTCL) [7]. Mycosis fungoides (MF) and Sezary syndrome (SS) represent the two most common subtypes of CTCL, accounting for the majority of cases [8]. PTCL can be classified into several different subtypes, among which include anaplastic large cell lymphoma (ALCL), angioimmunoblastic T cell lymphoma (AITL), extranodal natural killer (NK)-T cell lymphoma (ENKTL), enteropathy-associated T cell lymphoma (EATL), hepatosplenic T cell lymphoma (HSTCL), and PTCL-not otherwise specified (PTCL-NOS) which is the most common of the group [9, 10].

The overall prognosis for T cell malignancies varies depending on the type of disease, but in general is much poorer when compared to B cell malignancies. While the survival in T-ALL/LLy has significantly improved with the intensification of chemotherapy, there still remain very limited options for patients with relapsed/refractory disease [11–13]. ATLL remains a very challenging disease to treat, with a median survival of less than 12 months for the acute form of this disease [3–5]. Advanced stage CTCL has a median overall survival of 5 years [14, 15], whereas outcomes of PTCL vary depending upon the subtype, with ENKTL, EATL, and HSTCL having the poorest prognosis [9, 10]. While immunotherapy has revolutionized the treatment landscape of various cancers with the use of monoclonal antibodies, checkpoint inhibitors, bispecific T cell engagers, and chimeric antigen receptor (CAR) T cell therapy, only limited responses have been seen in T cell disease [15]. Some promising results have been seen with use of brentuximab vedotin, a CD30-directed immunotoxin, in CD30-positive PTCL and CTCL [16, 17] and the use of pembroluzimab, a programmed cell death receptor 1 (PD-1) inhibitor, in the treatment of ENKTL [18]; however, these positive results have been limited to very specific subsets of T cell disease. One form of immunotherapy that has not yet been successfully translated to T cell malignancies is that of chimeric antigen receptor (CAR)-based immunotherapy. CAR T cell therapy has been extremely successful in relapsed/refractory B cell malignancies as evidenced by the recent Food and Drug Administration (FDA) approval of two CAR T cell therapeutics for this disease [19–23]. However, implementing this technology to treat T cell malignancies has been difficult, primarily due to the lack of a tumor-specific surface antigen in cancerous T cells. In this review, we will discuss the challenges involved in translating this novel technology to T cell disease, review all the preclinical and clinical progress made in adapting this therapy for this challenging disease, and examine potential solutions for the future development of this innovative therapy.

## CAR T cell therapy

Genetic engineering of primary T cells was first presented in the late 1980s [24]. Since then, chimeric antigen receptor T cells have emerged as a promising technique for the treatment of relapsed/refractory malignancies. CAR therapy brings together numerous fields including immunology, tumor biology, genetic engineering, synthetic biology, and pharmacology. CARs are comprised of the intracellular signaling domain from the natural T cell receptor (TCR), CD3ζ, linked to a single-chain variable fragment (scFv) which serves as the antigen recognition domain. The scFv sequence is derived from a monoclonal antibody by combining the variable heavy (V_H_) and light (V_L_) domains using a small peptide linker. Commonly used CARs also include one or two costimulatory domains, such as CD28, 4-1BB, ICOS, and/or OX40. Although the kinetics have yet to be fully elucidated, it is essential that CAR T cells have mechanisms of trafficking to the tumor site where they can recognize their cognate antigen. This results in CAR T cell activation and expansion, and ultimately cytolytic activity against cells expressing the target antigen. CAR-based ligand recognition is advantageous compared to TCR-based ligand recognition because CAR-targeting is not restricted by major histocompatibility complex (MHC) interactions. Therefore, CARs can recognize cell surface proteins that have not been processed and presented by antigen presenting cells (APCs). Importantly, the interactions between scFvs and ligands have much higher affinity and avidity compared to that of TCR-ligand interactions [25]. Furthermore, the immune synapse formed from the interaction between a CAR and its ligand likely results in a much greater functional avidity than is observed using a targeted antibody approach with the same antibody (25).

CARs targeting the B cell antigen CD19 have been studied extensively for the treatment of B cell malignancies. In 2017, the FDA approved the first CAR T cell therapy, Kymriah, a CD19-directed CAR therapy for the treatment of relapsed/refractory B cell acute lymphoblastic leukemia (B-ALL) and in 2018, Yescarta was approved to treat relapsed diffuse large B cell lymphoma (DLBCL). These therapies, including others in clinical trials, have been widely successful in eliminating malignant cells and re-inducing remission in patients who were otherwise treatment-refractory [19–21, 26, 27]. Patients receiving CAR therapy undergo leukapheresis resulting in the collection of T cells, which are subsequently modified using a lentiviral or retroviral vector to express the CAR. These cells are expanded ex vivo while the patient undergoes lymphodepletion, a process involving chemotherapeutic agents. Finally, the CAR T cells are re-infused into the patient [28]. Lymphodepletion prior to re-infusion of the autologous T cells has been shown to augment both CAR T cell proliferation as well as persistence [29–31]. The administered dose of CAR T cells and the pre-existing tumor burden do not appear to be the sole determinants of the degree of T cell expansion, engraftment, and overall response. Other factors may be involved, such as the density of cognate antigen expression on the cancer cells [32]. However, the optimal degree of persistence of CAR T cells required to prevent leukemic relapse has not been determined [25, 33].

One of the mechanisms of relapse post-CD19 CAR T cell therapy is due to surface antigen escape with relapsed leukemia cells being CD19-negative. The mechanism may be due to the expansion of a small subset of CD19-negative cancer cells or alternatively, the cells may downregulate CD19 from the cell surface in order to evade detection by CAR T cells, rendering them resistant [19, 21, 34–37]. Additionally, it was recently shown that a phenomenon referred to as trogocytosis is a mechanism of antigen escape whereby the antigen is transferred to the CAR T cell [38]. It has also been shown that transduction of a single leukemic blast with an anti-CD19 CAR that was re-infused into a B-ALL patient, ultimately resulted in relapse and death of the patient [39]. Transduction of the leukemic cell resulted in masking of the target antigen through interactions between the CAR and the cognate antigen on the same cell. Clonal expansion of this population resulted in resistance to CAR therapy. This report emphasized the importance of strict and perfect isolation of normal, healthy T cells for modification with the CAR construct. As we discuss below, this is particularly challenging in T cell leukemia patients who are more likely to have circulating cancerous T cells, and therefore have a higher probability of these cells being inadvertently isolated, transduced, and re-infused.

Of note, there are severe toxicities that have been associated with CAR therapy. Cytokine release syndrome (CRS) is a systemic inflammatory response directly resulting from robust T cell activation following infusion. IL-6 is one pro-inflammatory cytokine that is secreted at high levels during CRS. During a particularly severe CRS condition, tocilizumab, an IL-6R antagonist monoclonal antibody, was used to rapidly and effectively reverse the symptoms of a pediatric patient [27]. Tocilizumab has since been FDA approved for treatment of CAR T cell-induced life-threatening CRS [40]. Neurological toxicities have been reported following CAR T cell infusion as well; however, preventative approaches remain elusive [36, 41–44]. Compared to CRS and neurotoxicity, a much more manageable consequence of CAR T cell therapy targeting B cell malignancies is the resulting B cell aplasia. This is a potentially lifelong outcome due to memory cell formation against a B cell antigen; but currently is managed by periodic infusions of intravenous immunoglobulins. Unfortunately, this is an extremely problematic outcome for T cell malignancies, as persistent T cell aplasia would be life threatening. There are currently > 200 clinical trials using CAR T cells registered at clinicaltrials.gov being carried out in the USA. However, the majority of these trials are enrolling patients with B cell malignancies. Advances are being made to expand CAR T cell therapy to the treatment of other cancers, and to minimize toxicities associated with treatment while reducing difficulty and cost of production.

## Translating CAR T cell therapy for treatment of T cell malignancies

**Fig. 1:**
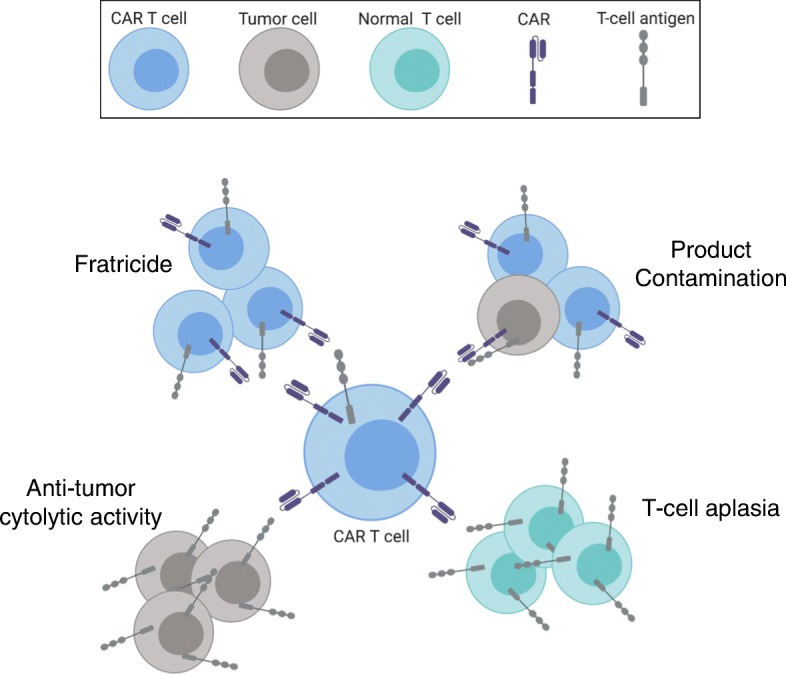
Potential outcomes of CAR T cell therapy in a patient with T cell disease. Upon re-infusion into a patient, CAR T cells recognize their cognate antigen, expanding upon this recognition, and initiating an attack. However, due to shared antigen expression on CAR T cells, normal T cells, and tumor cells, numerous outcomes can be observed. CAR T cells target tumor cells as intended, reducing tumor burden. However, without further engineering, the CAR-modified T cells are likely to express the targeted antigen as well, resulting in fratricide. CAR T cells would also target healthy T cells, resulting in unintended T cell aplasia. Lastly, CAR T cell therapy involves isolating normal T cells from malignant T cells for CAR-modification. A single malignant cell contaminating this population can result in masking of the antigen, leading to antigen-positive relapse. *Figure was created using BioRender

Harnessing and redirecting the cytotoxicity of T cells to malignant B cells has been established, but reprogramming T cells to kill malignant T cells, while sparing normal T cells, is much more complex and challenging. This requires aberrant expression of an antigen on malignant T cells that is absent or expressed at very low levels on normal T cells. CAR therapy requires isolation of healthy T cells from malignant T cells, a complicated procedure that can result in product contamination and subsequent CAR-modification of tumor cells. Additionally, expression of the targeted antigen on CAR T cells results in fratricide and limited expansion of the CAR T cells. Furthermore, targeting of an antigen regularly expressed on normal T cells would result in T cell aplasia, leading to profound immunosuppression, likely to be associated with high rates of morbidity and mortality (Fig. 1).

Various approaches have been used to overcome these challenges, including CRISPR-Cas9 genome editing to remove the antigen from the CAR T cells [45–47], Tet-OFF expression system to limit fratricide during ex vivo expansion [48], protein expression blocker (PEBL) to retain the antigen in the ER/Golgi to prevent cell surface expression [49, 50], or using CAR-modified natural killer cells instead of T cells [47, 51–54]. Additionally, to date, four targets have been investigated as targets for CAR T cell therapy for the treatment of T cell malignancies with limited to no expression in the normal population of T cells, CD30, CD37, TRBC1, and CD1a [55–58]. Table 1 provides a summary of potential solutions to the three main challenges seen in adapting CAR technology for T cell malignancies—fratricide, T cell aplasia, and product contamination. A list of all current CAR-based clinical trials targeting T cell disease is presented in Table 2. Below, we review all preclinical and clinical CAR studies targeting T cell malignancies categorized according to the target antigen of interest.

**Table 1:** Strategies to overcome challenges in translating CAR therapy to treat T cell malignancies

Challenge	Strategy	Reference
Fratricide	Targeting downregulated antigens (e.g., CD5)	[59]
	Genome editing of target antigen	[45–47]
	Targeting antigens with limited expression on T cells (e.g., CD30, CD37, TRBC1, CD1a)	[55–58]
	Tet-OFF expression system	[48]
	Protein expression blockers (PEBLs)	[49]
	Using NK cells or NK-92 cells	[47, 51–54, 60]
T cell aplasia	Targeting antigens with limited expression on T cells (e.g., CD30, CD37, TRBC1, CD1a)	[55–58]
	mRNA electroporation	
	Adeno-associated viral (AAV) vector delivery	
	Using NK cells or NK-92 cells	[47, 51–54, 60]
	Using γδ T cells	
	Suicide genes and safety switches	
	Bridge to allogeneic hematopoietic stem cell transplant (HSCT)	
Product contamination	Allogeneic CAR T cells with TRAC locus editing	[46, 61]
	Using NK cells or NK-92 cells	[47, 51–54, 60]
	Using γδ T cells

**Table 2:** Clinical CAR trials targeting T cell malignancies

T cell antigen	Clinical Trials	Sponsor	CAR costimulatory domain	Additional intervention	Phase	Status	Ref
CD5	NCT03081910 (MAGENTA)	Baylor College of Medicine	CD28	None	Phase I	Recruiting	
CD7	NCT04004637	PersonGen BioTherapeutics			Phase I	Recruiting	
	NCT04033302	Shenzhen Geno-Immune Medical Institute			Phase I/II	Recruiting	
	NCT03690011	Baylor College of Medicine	CD28	CRISPR/Cas9 CD7-editing	Phase I	Not yet recruiting	
	NCT02742727	PersonGen BioTherapeutics	CD28 and 4-1BB	NK-92 cells	Phase I/II	Unknown	
CD4	NCT03829540	Stony Brook University	CD28 and 4-1BB		Phase I	Recruiting	
CD30	NCT01192464	Baylor College of Medicine		EBV-specific CTL	Phase I	Active, not recruiting	
	NCT03383965	Immune Cell Inc	2^nd^ generation		Phase I	Recruiting	
	NCT02690545	UNC Lineberger Comprehensive Cancer Center			Phase I/II	Recruiting	[62]
	NCT02259556	Chinese PLA General Hospital	4-1BB		Phase I/II	Recruiting	[63]
	NCT02958410	Southwest Hospital, China			Phase I/II	Recruiting	
	NCT03049449	NCI			Phase I	Recruiting	
	NCT01316146	UNC Lineberger Comprehensive Cancer Center	CD28		Phase I	Active, not recruiting	[55]
	NCT02917083 (RELY-30)	Baylor College of Medicine	CD28		Phase I	Recruiting	[64]
	NCT04008394	Wuhan Union Hospital, China	3^rd^ generation		Phase I	Recruiting	
	NCT03602157	UNC Lineberger Comprehensive Cancer Center		CCR4 overexpression	Phase I	Recruiting	
	NCT02663297	UNC Lineberger Comprehensive Cancer Center	CD28		Phase I	Recruiting	
TRBC1	NCT03590574	Autolus Limited		RQR8 safety mechanism	Phase I/II	Recruiting	

### CD5

CD5 expression is limited to normal T cells and a small subpopulation of B cells, called B-1a cells [65–69]. CD5 acts as a negative regulator of TCR signaling and has a role in protecting against autoimmunity [70, 71]. CD5 is highly expressed on many T cell malignancies, particularly T-ALL and PTCLs, rendering it a good target for CAR T cell therapy [72–74]. Since CD5 expression on T cells is approximately ten times that on B cells [75], a low-affinity, high-avidity CAR targeting CD5 may steer clear of CD5-positive B cells while selectively killing T cells [76, 77]. Furthermore, CD8^+^ tumor-infiltrating lymphocytes (TILs) express lower levels of CD5 compared to that of peripheral blood T cells, and one study showed downregulation of CD5 improves the ability of T cells to lyse malignant cells [78]. CD5 was previously targeted as a tumor antigen in clinical trials using immunotoxin-conjugated CD5 monoclonal antibodies, with responses seen in patients with cutaneous T cell lymphoma and T-ALL [79, 80].

A preclinical study showed that expression of a CD5-CAR with a CD28 costimulatory domain resulted in surface downregulation of CD5 in CAR T cells. As a result, fratricide was observed only transiently, allowing the CD5-CAR T cells to expand. These cells had significant in vitro cytotoxicity against two T-ALL cell lines and primary T-ALL cells and delayed leukemia progression in two different CD5-positive T-ALL models [59]. Based on these results, CD5-CAR T cells with a CD28 costimulatory domain are being tested in patients with relapsed or refractory T cell disease (MAGENTA trial, NCT03081910). Our group used CRISPR-Cas9 to knockout CD5 expression in primary T cells prior to transduction with the CD5-CAR. We showed that gene editing of CD5 in effector CAR T cells increased CAR surface expression and decreased self-activation [47]. The increased CAR surface expression is predicted to enhance CAR T cell anti-tumor efficacy. We also showed antagonism of vasoactive intestinal peptide (VIP) signaling in conjunction with inhibition of the PI3Kδ pathway increased expansion of CD5-CAR-modified T cells as well as their cytotoxicity against CD5-specific tumor cell lines. This combination of compounds was also demonstrated to prolong in vivo persistence of treated T cells in NOD *scid* IL2Rγ-chain knockout (NSG) mice [81].

Interestingly, use of 4-1BB as the costimulatory domain in a CD5-CAR resulted in a significant fratricidal effect [48]. It was shown that tumor necrosis factor (TNF) receptor-associated factor (TRAF) signaling from the 4-1BB endodomain upregulated the intercellular adhesion molecule 1 (ICAM1), which subsequently stabilized the fratricidal immunological synapse between CD5-CAR T cells containing the 4-1BB costimulatory domain. To limit and control the effects of fratricide, a Tet-OFF expression system was used, which allowed for controlled transgene expression using the small molecule inhibitor, doxycycline. In the presence of doxycycline, CD5-41BB-CAR T cells expanded ex vivo without evidence of fratricide, while maintaining a more naïve genotype. Doxycycline was removed from the culture prior to injecting the CD5-41BB-CAR T cells into mice, resulting in CD5-CAR expression and improved survival outcomes in a T-ALL mouse model. Furthermore, there was a survival advantage in mice treated with Tet-OFF CD5-41BB-CAR T cells compared to survival of mice treated with CD5-CD28-CAR T cells without the Tet-OFF expression system [48].

Alternatively, we expressed the CD5-CAR in NK-92 cells, an interleukin-2 (IL-2) dependent natural killer cell line, which are inherently CD5-negative. Our data demonstrates that CD5-CAR-modified NK-92 cells have increased cytotoxicity against T cell leukemia cell lines compared to the cytotoxicity of naïve NK-92 cells [47, 51], and there is a significant improvement in survival of T-ALL xenograft mouse models compared to survival of mice treated with naïve NK-92 cells [47]. This data confirms previously published data illustrating significantly improved survival and enhanced tumor reduction in irradiated T-ALL mouse models treated with CD5-CAR-modified NK-92 cells compared to that of mice treated with control NK-92 cells [53]. Recently, another group tested CD5-CAR-modified NK-92 cells, using a NK-specific costimulatory domain 2B4 in their CAR constructs [82]. Interestingly, the CD5-2B4-CAR NK-92 cells displayed superiority to CD5-41BB-CAR NK-92 cells, in both in vitro and in vivo experiments [82].

### CD7

CD7 is a transmembrane glycoprotein with expression on T cells and NK cells [83]. The majority of T-ALLs are CD7-positive, despite some populations lacking expression of other common markers, such as the TCR [74, 84]. Additionally, early T cell precursor acute lymphoblastic leukemia (ETP-ALL), a high-risk subset of T-ALL, highly express CD7 [84–86]. Two clinical trials have been initiated in China studying CD7-CAR-modified T cells for the treatment of CD7-positive malignancies (NCT04033302 and NCT04004637). However, preclinical studies showed significantly reduced expansion of CD7-CAR T cells compared to control T cells, as a result of fratricide [45, 49]. Fratricide appears to be observed to a greater extent in CD7-CAR T cells compared to CD5-CAR T cells [45]. It is hypothesized that this is due to a more incomplete internalization mechanism of CD7 from the cell surface following ligation of the antigen with an anti-CD7 scFv. CRISPR-Cas9 editing of CD7 from the cell surface of T cells prior to CAR expression demonstrated a superior method of developing CD7-CAR T cells. These cells exhibited limited fratricide, expanded in vitro, and showed no evidence of impaired cytotoxicity in vitro nor in vivo. Investigations in a T-ALL mouse xenograft model revealed a statistically significant prolonged survival of CD7-edited CD7-CAR-treated mice compared to survival of control mice [45]. Based on these results, a phase I clinical trial has been initiated testing CD7-CD28-CAR T cells in T-ALL patients (NCT03690011). Additionally, a UCART7 was generated using CRISPR-Cas9 genome editing to disrupt the CD7 and TCRα constant (TRAC) loci. This study demonstrated that NSG mice engrafted with primary T-ALL blasts and treated with UCART7 donor cells exhibited tumor clearance from the peripheral blood, and, did not develop graft versus host disease (GvHD) or other severe side effects [46].

A new technique using protein expression blockers (PEBLs) has been established as an alternative to genome editing. This strategy couples an scFv with a retention peptide to maintain the protein of interest in the ER/Golgi preventing cell surface expression of the antigen. PEBL-CD7-CAR T cells exhibited superior cytotoxicity against primary T-ALL cells in vitro compared to non-PEBL CD7-CAR T cells. Using a patient-derived xenograft (PDX) model of ETP-ALL, upon detection of leukemic cell expansion in peripheral blood, PEBL-CD7-CAR T cells were injected. PEBL-CD7-CAR T cell-treated mice had a significant survival advantage over control mice. However, CD7-positive relapse did occur in all PEBL-CD7-CAR T cell-treated mice [49].

Despite CD7 expression on NK-92MI cells (IL-2 producing NK-92 cells), they have been used for CD7-CAR therapy demonstrating only a small percentage of cells are CD7-positive, and upon CD7-CAR expression, fewer than 1% CD7-positive NK-92MI cells remain [60]. Two CD7-CAR constructs, a monovalent and bivalent construct, were generated using a humanized CD7 nanobody sequence that had been previously developed in the laboratory. Both CAR constructs demonstrated enhanced CD7-specific cytotoxicity against T-ALL cell lines and primary patient cells ex vivo when expressed in NK-92MI cells. The bivalent CD7-CAR-modified-NK-92MI cells exhibited slightly greater cytotoxicity compared to that of the monovalent CAR-modified cells, and significantly inhibited disease progression in a T-ALL PDX model when compared to naïve unmodified NK-MI cells.

### CD4

Most cancers derived from lineage-differentiated T cells are likely to be of CD4-positive origin, making CD4 a potential target for CAR therapy. A preclinical study was performed to consider the cytotoxicity of CD4-CAR-modified T cells against T-ALL tumors in NSG mice. This study also included the use of alemtuzumab to clear the CAR T cells as a safety mechanism. NSG mice were injected with luciferase-expressing Jurkat T cells and subsequently treated with naïve T cells or CD4-CAR-modified T cells. CAR-treated mice displayed a survival advantage and an ~ 80% reduction in tumor burden compared to mice treated with naïve T cells. CD4-CAR-modified T cells were also injected into mice to evaluate the ability of alemtuzumab to effectively eliminate CAR-modified T cells. Alemtuzumab was administered 24 h post-CAR T cell injection. A > 95% depletion of CD4-CAR-modified T cells was observed within 6 h following injection signifying the use of alemtuzumab as a safety mechanism for CAR T cell therapy [87]. Additionally, a phase I clinical trial to assess the safety and feasibility of CD4-CAR T cell infusions in patients with relapsed/refractory T cell lymphoma and T cell leukemia has been initiated (NCT03829540).

However, expression of CD4 on T cells can complicate CD4-CAR T cell therapy as previously described. NK-92 cells are inherently CD4-negative, and therefore the use of NK-92 cells as opposed to T cells reduces risk of fratricide and avoids the need for further modifications. Additionally, it abrogates the risk of aplasia of CD4-positive cells that can occur with long-term engraftment of CAR T cells. Anti-CD4-CAR NK-92 cells have shown in vitro success eliminating PTCL cell lines and both adult and pediatric primary cells. Using a xenograft model in NSG mice, CD4-CAR NK-92 cell-treated mice demonstrate significantly prolonged survival compared to control-modified NK-92 cell-treated mice [54].

### CD37

CD37 is a member of the tetraspanin superfamily with expression limited to lymphoid tissues, particularly B cells [88, 89]. CD37 expression in cancer cells is typically characteristic of B cell malignancies; however, its expression can be found in some cases CTCL and PTCL [90, 91]. Since CD37 is not expressed in T cells, there is no evidence of fratricide occurring in anti-CD37 CAR T cells. However, in the presence of CD37-positive PTCL cell lines, CD37-CAR T cells exhibit increased activation and degranulation as well as specific cytolytic activity in vitro [56]. The restricted expression of CD37 makes it a safer target for CAR T cell therapy, given there would be no concern of T cell aplasia. Additionally, CD37 is not expressed in NK cells, providing an opportunity to utilize NK cells as effector cells in place of T cells. The versatility of CD37-CARs to treat B cell and T cell lymphomas suggests that this may be an important target for further investigations. While CD37 is predominantly being examined for dual targeting for B cell malignancies, the target has potential for CAR therapy against T cell malignancies.

### CD30

CD30, a member of the tumor necrosis factor receptor (TNFR) superfamily, promotes T cell proliferation and cytokine production following TCR stimulation, while also having an opposing role in promoting apoptosis [92]. Expression is limited to a subset of activated lymphocytes found around the follicular regions of lymphoid tissues [93–95]. While CD30 is well known for its strong expression in virtually all classical Hodgkin lymphoma, expression of CD30 can also be found on a subset of PTCLs, including ALCL [92–94, 96]. One study demonstrated that CD30 expression is upregulated during chemotherapy regimens in T-ALL patients. Of 34 T-ALL patients, approximately 38% had CD30-positive T-ALL [96]. Therefore, some T-ALL patients who relapse following chemotherapy may still respond to CD30-directed CAR therapy.

Preclinical studies have previously demonstrated CD30-CAR T cell capacity for lysing tumor cells [97, 98] and numerous clinical investigations into CD30-CAR T cell therapy have been launched with encouraging results. Eleven phase I/II trials treating patients with CD30-positive malignancies are currently active (NCT01316146 [55], NCT01192464, NCT03049449, NCT02690545 [62], NCT02958410, NCT02663297, NCT03383965, NCT02917083 [64], NCT04008394, NCT02259556 [63], and NCT03602157). To date, no toxicities related to CAR T cell infusion nor impaired immunity against common viruses has been reported from these trials. However, one trial reported that the in vivo CAR T cell expansion and persistence was reduced following subsequent infusions compared to those following initial doses [55]. The decreased persistence of the CAR T cells may have prevented the development of severe adverse events such as CRS and neurotoxicity that are commonly observed following CAR T cell infusion. Of the two ALCL patients in this trial, one patient was non-responsive to the therapy, while the other entered complete remission lasting 9 months [55]. Results from another phase I trial in China for patients with relapsed/refractory CD30-positive lymphomas (NCT02259556) corroborate the limited toxicity and anti-tumor activity of CD30-CAR T cells [63].

### TRBC1

T cells express the αβ TCR; the β-chain can either be encoded by the T cell receptor beta constant 1 (TRBC1) gene or TRBC2 gene [99, 100]. Therefore, expression of TRBC1 and TRBC2 is mutually exclusive. Additionally, CD4- and CD8-positive T cell populations express both subsets and CD8-positive T cell populations specific for common viruses also contain both TRBC1 and TRBC2 cells [58]. However, as malignant T cells develop from a single cell, the entire population of cancerous cells will be either TRBC1- or TRBC2-positive. Numerous T cell malignancy cell lines and primary samples have been analyzed by flow cytometry to validate the homogeneity of β-chain expression in a malignant cell population [58]. Many cancer cells downregulate the αβ TCR; however, it is expressed on > 95% of PTCLs [101] and > 30% of T-ALLs [102].

Anti-TRBC1 CAR T cells exhibited specific and efficient cytotoxicity against the JKO T cell line transduced with TRBC1, but not against non-transduced cells or cells transduced with TRBC2, even in a mixed population. Furthermore, in primary samples from patients with T cell malignancies, the anti-TRBC1 CAR T cells preserved a significant fraction of healthy T cells (TRBC2 cells), thereby circumventing a limitation of CAR T cell therapy for the treatment of T cell malignancies [58]. In an NSG mouse model using TRBC1-positive Jurkat T cells to establish cancer, mice treated with the anti-TRBC1 CAR T cells exhibited reduced tumor burden and elongated survival. In additional preclinical studies, NSG mice were injected with both TRBC1 and TRBC2 cancer cells, and then treated with either naïve T cells or anti-TRBC1 CAR T cells. TRBC1-positive Jurkat T cells could not be detected in mice treated with anti-TRBC1 CAR T cells; however, TRBC2-positive cells were identified. This is in contrast to mice treated with naïve T cells, whose bone marrow confirmed the presence of both TRBC1-positive and TRBC2-positive cells [58]. Thus, targeting TRBC1-positive malignant cells offers a unique approach to avoiding T cell aplasia, a consequence of many proposed CAR T cell therapies for the treatment of T cell malignancies.

### CD3

CD3 is a pan T cell marker comprised of four distinct polypeptide chains, epsilon, gamma, delta, and zeta, which form pairs of dimers, transmitting T cell activation signals. As CD3 is exclusively expressed on T cells, it has been a popular target in preclinical CAR T cell therapies for the treatment of T cell malignancies. As expected, due to fratricidal issues, manufacturing of anti-CD3 CAR T cells does not yield a viable cellular product [61]. Various approaches using an anti-CD3 CAR have been investigated including the use of transcription activator-like effector nuclease (TALEN) mRNA to disrupt the TRAC locus and using NK-92 cells in place of T cells as the effector cell type. Disruption of the TRAC locus prevents assembly of the TCRαβ/CD3 complex, allowing for anti-CD3-CAR expression without compromising cellular proliferation and viability. Enrichment of the CAR-positive, CD3-negative population was observed. In patient T-ALL samples, anti-CD3 CAR T cells demonstrated specific cytotoxicity against CD3-positive cells. In a T-ALL NSG model, anti-CD3 CAR T cells were shown to clear luciferase-expressing CD3-positive Jurkat cells, but showed no effect in NSG mice engrafted with CD3-negative Jurkat cells [61]. To circumvent the need for additional modifications, NK-92 cells can also be used to express the anti-CD3-CAR, since they are CD3-negative cells. CD3-CAR NK-92 cells demonstrated efficient ex vivo lysis of PTCL primary samples, resulting in less than 0.5% lymphoma cells remaining at 5:1 effector to target ratios. Furthermore, CD3-CAR NK-92-treated T-ALL NSG mice exhibited prolonged survival with ~ 87% reduced tumor burden through day 23 [52].

### CD1a

CD1a is a lipid-presenting molecule whose expression is restricted to developing cortical thymocytes, skin Langerhans cells, and some circulating myeloid dendritic cells [103, 104]. Neither T cells nor CD34^+^ hematopoietic progenitors express CD1a, making it a fratricide-resistant target, while limiting the risk of on-target/off-tumor toxicity. Expression in T cell malignancies is only limited to cortical T-ALL, a major subset of T-ALL accounting for ~ 35–40% of all T-ALL cases [105, 106]. A study showed that CD1a-CAR T cells expanded without fratricide, and had long-term persistence in an in vivo model [57]. Additionally, these cells demonstrated specific cytotoxic activity against CD1a-positive T-ALL cell lines and primary blasts in vitro, and exhibited potent anti-leukemic activity in a PDX model of cortical T-ALL. Thus, while not applicable to all T cell malignancies, targeting CD1a with CAR T cells may be successful in the specific subset of cortical T-ALL cases.

## “Off-the-shelf” CAR T cell therapy

One of the greatest challenges in utilizing autologous CAR T cell therapy for the treatment of T cell malignancies is the separation of healthy T cells from malignant T cells, in order to generate a CAR T cell product that is not contaminated with cancerous T cells. To date, there has been one reported case from the University of Pennsylvania of CD19-CAR modification of a single leukemic B cell, resulting in CD19-positive relapse and ultimately death of the patient [39]. This task of isolating healthy T cells is even more difficult when a proportion of the patient’s T cells are malignant, especially in cases of T cell leukemia where there is a high likelihood of circulating cancerous T cells. Thus, manufacturing of autologous CAR T cells for the treatment of T cell malignancies has a very high likelihood of resulting in CAR-modified leukemic cells. This would likely result in relapse as these cells would likely escape recognition by normal CAR-T cells.

Additionally, there remain numerous challenges to using a patient’s own cells to manufacture CAR T cells. Patients with advanced disease undergoing CAR T cell therapy typically are heavily pre-treated, having previously undergone numerous rounds of chemotherapy, which can result in low T cell counts and/or T cells that may not be healthy enough to expand well making it very difficult to manufacture an efficacious CAR T cell product [107]. This issue is much more prevalent in adult patients due to the decreasing proportion of naïve T cells associated with aging [107–110]. Additionally, given that many of these patients have advanced disease, a patient may experience disease progression, co-morbidities, or even death in the time it takes to manufacture autologous CAR T cells. This is especially true in most relapsed T cell malignancies, which tend to be aggressive and chemo-resistant in nature. Lastly, each starting autologous T cell product is different—variable function, maturation, CD4/CD8 ratios, and phenotypic ratios—and the heterogeneity of each individual product has led to unpredictable results and variable potency of the therapy.

An alternative to autologous CAR T cell manufacturing is the use of allogeneic T cells as the cell source. In order to make this approach feasible, expression of the endogenous αβTCR in allogeneic CAR T cells must be blocked as it would likely result in GvHD, unless the donor is a human leukocyte antigen (HLA) match. This process involves leukapheresis from a healthy donor, followed by isolation of the donor’s T cells. Following transduction of the T cells with a CAR-encoding retroviral vector, subsequent genome editing of the TRAC locus is required to prevent expression of the endogenous TCR. Cells that remain TCR-positive are then depleted from the expanded CAR T cell product prior to cryopreservation. This creates an “off the shelf” cellular product that can be banked until it is needed for therapy. This approach resulted in successful remission in two infant B-ALL cases treated with allogeneic CD19-CAR T cells modified at the TRAC and CD52 loci. The allogeneic CAR T cells persisted until conditioning for stem cell transplant [111]. Another group utilized shRNA to knock down β2-microglobulin in conjunction with a knock-in strategy to insert a CD19-CAR into the TRAC locus. Knock down of β2-microglobulin reduces the ability of class I HLA molecules to form heterodimers on the cell surface. Reducing expression of both β2-microglobulin and TRAC resulted in decreased allogeneic attack by CD8 T cells and NK cells [112]. This strategy may be useful to reduce allo-recognition in patients receiving CAR T cell therapy. Other groups have exploited similar approaches in preclinical CAR T cell investigations targeting CD7 and CD3, as previously described [46, 61].

CRISPR-Cas9 genome editing has become a popular technique to prevent gene expression or to correct gene expression. One study targeting CD7 generated “fratricide resistant, allo-tolerant” CAR T cells using CRISPR-Cas9 to disrupt both CD7 and the TRAC loci (UCART7). NSG mice engrafted with primary T-ALL blasts developed GvHD when treated with wildtype donor T cells; however, mice treated with UCART7 donor cells were able to clear the tumor cells from the peripheral blood, and, furthermore, did not develop GvHD or other severe side effects [46]. TALENs, an alternative genome editing technique, have also been used to prevent expression of the TRAC locus in order to limit fratricide of anti-CD3-CAR T cells and prevent MHC-recognition of foreign host cells. Genome editing the TRAC locus prevents stable assembly of the TCRαβ/CD3 complex. Disruption of the TRAC locus using TALEN mRNA prior to transduction with an anti-CD3-CAR lentiviral vector yielded CAR T cells that proliferated well and greatly reduced tumor burden in an NSG mouse model of human leukemia [61].

As described above, PEBLs have been recently developed to selectively prevent expression of individual proteins. PEBLs have been shown to effectively retain CD3ε in the ER/Golgi to prevent MHC recognition of host cells during allogeneic use of anti-CD19 CAR T cells [50]. Disruption of TCRαβ signaling had no effect on T cell proliferation. There was no evidence of GvHD in an NSG mouse model of leukemia treated with the PEBL-CD19-CAR T cells, whereas 60% of the mice treated with CAR T cells that were not expressing the CD3ε PEBL developed GvHD. Furthermore, both PEBL and CAR can be expressed from the same vector using a 2A sequence, resulting in only one transduction of the cells [50]. While this study utilized PEBL in conjunction with an anti-CD19-CAR, this system can potentially be applied with other CAR constructs to target T cell antigens.

### Alternative effector cell types

While CAR-modified αβ T cells can have a memory phenotype resulting in T cell aplasia, NK cells and gamma delta (γδ) T cells will not. Utilizing these innate cells for CAR therapy is a viable alternative that groups are exploring. One disadvantage to preventing memory cell formation and using effector cells with limited persistence is reduced tumor control. However, this limitation can potentially be overcome by utilizing these cells in multiple dosing regimens. Repeated dosing of short-lived CAR-expressing cells can be used to induce remission; thus, providing a bridge to an allogeneic hematopoietic stem cell transplant (HSCT) if needed. Since these products would be utilized in an allogeneic setting, they can be cryopreserved and would be readily available when needed for use.

### Natural killer cells and NK-92 cells

Ex vivo-expanded NK cells are short-lived, and do not persist for extended periods of time in vivo compared to that of αβ T cells [113]. CAR-modified NK cells have a turnover time of 1–2 weeks; therefore, there is reduced concern of aplasia of antigen-expressing cells [114]. Currently, there are two active clinical trials using anti-CD19-CAR-modified NK cells (NCT00995137 and NCT01974479). Additionally, some studies use NK-92 cells, an IL-2-dependent NK-lymphoma-derived cell line. NK-92 cells are often used as an alternative to primary NK cells due to their ease of expansion under current good manufacturing process (cGMP) conditions [115] and transfection with CAR mRNA [116]. CAR-modified NK or NK-92 cell infusion can result in tumor cell clearance without the risk of GvHD. Therefore, these cells typically only require one genetic modification. Additionally, with the exception of CD7, NK cells do not express antigens targeted in T cell malignancies. Therefore, neither fratricide nor T cell aplasia are of primary concern.

CAR-expressing NK-92 cells have been extensively assessed in preclinical studies targeting various cancers such as B cell malignancies [117–119], multiple myeloma [120], acute myeloid leukemia (AML) [121], breast carcinoma [122, 123], neuroblastoma [124], and glioblastoma [125]. As previously discussed, multiple groups have initiated preclinical studies using CAR-modified NK-92 cells for the treatment of T cell malignancies, targeting antigens such as CD5, CD7, CD4, and CD3, demonstrating reduced tumor burden and an overall survival benefit in NSG mouse models of T cell leukemia [47, 52–54, 60]. The safety and efficacy of NK-92 cells has been evaluated in clinical trials displaying a good safety profile with few mild to moderate adverse events [126–128] (NCT00900809, NCT00990717). To date, five clinical trials have been initiated involving infusion of CAR-modified NK-92 cells targeting a variety of antigens, including CD33 [129], human epidermal growth factor receptor 2 (HER2), B cell maturation antigen (BCMA), CD19, and the T cell antigen, CD7 (NCT02944162, NCT03383978, NCT03940833, NCT02892695, and NCT02742727).

Inherent NK-cell cytotoxicity is dependent on the balance of activating and inhibitory killer-cell immunoglobulin-like receptor (KIR) signals. Inhibitory and activating KIRs on NK cells form a balance, as there are often signals from both inhibitory and activating receptors. The inhibitory signals predominate, typically through higher affinity for their ligands; however, strong activating signals can override the inhibitory signals, licensing NK cells to kill. If donor inhibitory KIRs do not recognize patient HLA, there is reduced inhibitory signaling to counteract the activating signaling [130, 131]. While NK-92 cells lack many of the inhibitory KIRs expressed on primary NK cells, they have a wide range of activating receptors [132]. Similar to NK cells, NK-92 cells have the capability to produce perforin and granzyme upon activation, as well as display cytotoxic activity through upregulation of TNF-related apoptosis-inducing ligand (TRAIL), Fas ligand (FasL), and TNFα [133]. Additionally, NK-92 cells have demonstrated evidence of serial killing, with each cell killing numerous target cells [134]. However, as NK-92 cells were derived from a NK cell lymphoma, they require irradiation prior to infusion into a patient to prevent expansion, resulting in persistence for about 1 week in vivo and potentially exhibiting reduced cytotoxicity. Alternatively, suicide mechanisms can be engineered into the cells to eliminate the risk of NK-92-cell persistence in vivo and eliminate the need for irradiation, thereby resulting in greater cytotoxicity of the infused cells.

NK cells exhibit their cytotoxic activity through numerous means, including expression of FasL or TRAIL, secretion of perforin and granzyme, as well as through antibody-dependent cellular cytotoxicity (ADCC) mechanisms [131, 135, 136]. A major limitation to the use of CAR T cells is antigen escape; however, as NK cells can kill through other mechanisms, downregulation of the cognate antigen on tumor cells may not halt anti-tumor activity. NK cells also express the natural killer group 2D (NKG2D) receptor, which recognizes cellular stress ligands such as MHC class I chain-related protein A/B (MICA/B) and UL16 binding proteins (ULBPs) [137, 138], resulting in cytotoxicity against exceedingly stressed cells. As NK cells do not recognize targets on healthy cells, they have limited off-target toxicity [131]. Additionally, their serial killing capability allows each individual NK cell to kill, on average, four tumor cells [139]. However, NK cells are notoriously difficult to expand ex vivo, transduce with viral vectors, cryopreserve, and they have limited life span in vivo [128, 140]. While autologous NK cells can be obtained by leukapheresis followed by selection of CD56-positive cells, allogeneic NK cells derived from a third party donor requires an additional step for depletion of alloreactive T cells from the donor product [141].

Purification and expansion of NK cells from peripheral blood mononuclear cells (PBMCs) have been optimized in cGMP protocols to clinically relevant numbers [142–144]. This is a time-consuming process as only 10% of PBMCs are NK cells [145]. However, recently developed methods are being used to enhance NK-cell expansion, such as through K562-feeder cell expression of OX40 ligand [146]. As mentioned above, a limitation to CAR-NK therapy is the extreme sensitivity of NK cells to cryopreservation. They have demonstrated poor viability and diminished cytotoxicity after cryopreservation. While cytotoxicity can be restored to normal levels after a few days in culture with exogenous IL-2, the low viability post-cryopreservation remains a concern [141].

### Gamma delta T cells

While αβ T cells function as a part of the adaptive immune system, γδ T cells play roles in both the innate and the adaptive immune systems. γδ T cells and αβ T cells originate from two distinct T cell lineages [224]. γδ T cells are the only innate immune cells expressing a TCR [147]; however, their target recognition is independent of MHC recognition [148, 149]. Lack of MHCI- and MHCII-restriction make γδ T cells optimal candidates for allogeneic cell therapy. The peripheral blood subset of γδ T cells known as Vγ9Vδ2 T cells represents the most commonly studied subset in this context. Studies by our group have demonstrated that similar transduction efficiencies can be achieved in Vγ9Vδ2 T cells grown under cGMP serum-free conditions as are achieved in αβ T cells using lentiviral vectors. Additional studies were performed revealing peak low-density lipoprotein receptor (LDL-R) expression on days 6–8 of γδ T cell expansion [150]. As LDL-R is the major receptor for VSV-G-pseudotyped lentiviral vectors, this data suggests that greater transduction efficiency can be achieved on these days using lentiviral vectors compared to earlier or later in the expansion [151].

To date, numerous preclinical studies have evaluated CAR-modified γδ T cells targeting neuroblastoma [152, 153], melanoma [154], B cell malignancies [153, 155], and epithelial cell adhesion molecule (epCAM)-positive adenocarcinomas [156]. GD2-CAR-modified γδ T cells expressing the RQR8 suicide gene were shown to expand 2.5-fold upon antigen exposure [152]. Furthermore, both GD2-CAR- and CD19-CAR-modified γδ T cells were demonstrated to secrete pro-inflammatory cytokines in the presence of GD2- or CD19-expressing tumor cells, respectively [153]. While these studies utilized viral vectors to express the CAR, electroporation of a Sleeping Beauty transposon has also been shown to result in CD19-CAR expression in γδ T cells, resulting in anti-tumor cytotoxicity in both the in vitro and in vivo settings [155]. Additionally, expression of a CAR targeting melanoma-associated chondroitin sulfate proteoglycan (MCSP) was established in γδ T cells using mRNA transfection. Despite comparable anti-tumor cytotoxicity, lower cytokine secretion was observed in MCSP-CAR-modified γδ T cells compared to that from conventional CAR-modified αβ T cells [154]. Reduced pro-inflammatory cytokine secretion is favorable due to anticipated reduced severity of CRS. Lastly, epCAM CAR-modified γδ T cells demonstrated high levels of in vitro cytotoxicity of tumor cell lines when γδ T cells were both fresh and cryopreserved [156]. These studies pave the way for additional trials using CAR-modified γδ T cells targeting T cell malignancies. They demonstrate that engineering of γδ T cells is feasible and results in enhanced in vitro and in vivo cytotoxicity upon CAR expression.

CAR-modified γδ T cells may be able to overcome the obstacle of antigen escape seen in some treatment-resistant cases by relying on their innate ability to recognize tumor cells through other means. Naïve γδ T cells have been shown to have anti-tumorigenic activity against leukemia, neuroblastoma, and colon cancer cell lines as well as primary cancer cells in vitro [157–160]. They are found in peripheral blood, spleen, and lymph nodes, in addition to almost all mucosal tissues, functioning as immune-surveillance of epithelial tissues by scanning for inflammatory threats [161, 162]. The γδ TCR recognizes self-antigens that serve as endogenous danger signals such as heat shock proteins, which are upregulated in cells with increased metabolism, like cancer cells. Expression of scavenger receptors like the NKG2D receptor enables γδ T cell activation through the interactions with antigens of cellular stress such as MICA/B and ULBPs [147, 163–166]. Additionally, γδ T cells express chemokine receptors that can detect chemokines secreted by cancer cells, likely facilitating their migration toward the tumor site [167]. γδ T cells also express FasL (CD95L) as a means of recognizing Fas expression on tumor cells and initiating apoptosis [168].

Another mechanism by which γδ T cells recognize tumor cells is through stimulation by phosphoantigens, such as isopentenyl pyrophosphate (IPP), which are recognized by the γδ TCR. While there are many subsets of γδ T cells, phosphoantigens specifically expand the Vγ9Vδ2 subset. IPP is used as a substrate in the mevalonate pathway by farnesyl pyrophosphate synthase (FPPS). Bisphosphonates overproduced in cancer cells block FPPS, resulting in a buildup of IPP, which is subsequently recognized by cytotoxic Vγ9Vδ2 T cells [169–172]. Bisphosphonate stimulation of γδ T cells has been applied to in vitro expansion of γδ T cells in conjunction with IL-2 in serum-free conditions [150]. A preclinical study involving nude mice receiving repeated dosing of γδ T cells resulted in decreased tumor growth model; however, tumor growth resumed upon completion of the γδ T cell infusions [173]. In phase I clinical trials, adoptive transfer of γδ T cells to patients receiving ex vivo expanded γδ T cells with a combination of IL-2 and bisphosphonate stimulation demonstrated the safety of the infused product and suggested that the therapy could be efficacious in slowing the progression of the disease. However, mixed results were seen in terms of efficacy, suggesting that genetic modification with CAR expression is likely to be more beneficial compared to γδ T cell therapy alone [169, 174–177].

While the autologous transfer of CAR-modified αβ T cells targeting a T cell malignancy can be used as a bridge to transplant (although the risk remains that a single CAR T cell will be left behind ultimately resulting in the development of T cell aplasia), it cannot be a curative option unless a near perfect design of a suicide gene, switch mechanism, or another system has been implemented to reliably eliminate all CAR T cells upon completion of the treatment. Therefore, effector cells with a limited lifespan such as γδ T cells, NK-92 cells, or NK cells are likely to be more effective in targeting T cell disease. Other techniques such as mRNA electroporation or adeno-associated viral (AAV) vector delivery can also be useful in preventing long-term CAR T cell persistence, as described below.

## Prevention of memory cell formation and T cell aplasia

While current CAR T cell therapies for the treatment of B cell malignancies have been hugely successful in inducing and maintaining remission, these therapies have prevented the re-emergence of endogenous B cells in patients in whom the CAR T cells have persisted. The CAR T cells can have a memory phenotype that allows them to remain dormant until restimulation with the cognate antigen, CD19, expressed on all endogenous B cells. While B cell aplasia is an undesirable side effect of these therapies, it has been managed by continued periodic intravenous immunoglobulin injections [36]. The long-term implications of persistent B cell aplasia remain unknown. In contrast, treatment of T cell malignancies using CAR T cells targeting antigens expressed on the majority of normal T cells is predicted to result in T cell aplasia. While B cell aplasia is tolerable, there is no such treatment for T cell aplasia. Patients who develop T cell aplasia will have profound immunosuppression and can potentially succumb to deadly infections [36]. Therefore, prevention of memory cell formation of CAR T cells and subsequent T cell aplasia remains an essential challenge to translating CAR T cell therapy for the treatment of T cell malignancies. While bridging a patient to an allogeneic HSCT following CAR T cell therapy may eliminate the risk of life-threatening T cell aplasia by clearing out the CAR T cells, safer and less invasive alternatives must also be explored to downregulate CAR activity after tumor clearance.

### mRNA electroporation

There are numerous disadvantages to using retroviral vectors for CAR T cell therapy, including risk of clonal dominance [178, 179], high cost of production [180], maximum cargo size [181, 182], and the inability to “turn off” transgene expression and unpredictable integration sites potentially resulting in insertional oncogenesis [183, 184]. The indefinite period of CAR expression can result in severe on-target off-tumor toxicities, which is particularly challenging to manage in T cell disease. To overcome these unintended side effects, groups are alternatively exploring delivery of CAR mRNA through electroporation as a safer method [185–187]. As with the use of effector cells with limited persistence in vivo, therapies with transient CAR expression require multiple infusions into the patients. Use of mRNA electroporation of T cells for CD19-CAR expression has been reported in a preclinical model, demonstrating reduced tumor burden 1 day post-treatment. This study illustrated prolonged survival of a xenograft mouse model after a single injection of CAR mRNA T cells; however, as predicted, as the mRNA levels decreased the tumor burden increased [185]. Published results from the first non-viral CD19-CAR clinical trial using mRNA electroporation to deliver the CAR into T cells demonstrated the safety and efficacy of this treatment in four relapsed/refractory classical Hodgkin lymphoma patients [225]. CAR mRNA was detected 48 h post-infusion; however, no mRNA could be detected by day 21. While only transient responses were seen, no severe toxicities were observed using this approach. Utilizing this non-viral strategy in T cell disease can be particularly advantageous, as it prevents the risk of long-term T cell aplasia. While the transient efficacy precludes this approach from being used as a definitive treatment, it could potentially serve as an effective bridge to transplantation.

### Adeno-associated viral vector

AAV is an alternative viral delivery method that can overcome some of the disadvantages of using integrating viral vectors as previously discussed. AAV is a single-stranded, non-enveloped DNA virus with a cargo capacity of approximately 4.7 kilobases [188]. Upon deletion of the Rep protein, the viral transgene forms circular concatamers that exist episomally in the nucleus of the cell. AAV expression is therefore diluted upon each mitotic division, resulting in a transient transgene expression limited to the lifespan of the cell [189, 190]. Thus, AAV delivery can control the duration of CAR expression, which is a desired quality to regulate cytokine production and mediate toxicities [191–193]. In particular, transient CAR expression may prove to be advantageous in the setting of T cell malignancies, by preventing unintended T cell aplasia.

Efficient transduction of innate immune cells, such as NK cells and γδ T cells, by an AAV vector would be particularly invaluable in targeting this group of diseases. As previously discussed, both NK cells and γδ T cells are excellent candidates as CAR effector cells against T cell antigens. A common challenge reported in using these cell types is the low transduction efficiency using integrating viral vectors, delaying progress in the development of these therapies. AAV gene transfer of a CAR into innate immune cells would offer the opportunity to develop an allogeneic off-the-shelf CAR therapeutic that can control CAR expression, thereby mitigating CRS and other adverse events. Additionally, the lack of memory cell formation against T cell antigens in these cell types will completely negate the risk of T cell aplasia. The AAV capsid directs the infectivity of different tissues, and therefore the appropriate capsid serotype must be used to maximize transduction of the desired cell type [194, 195]. AAV6 has been shown to result in higher transduction of hematopoietic stem and progenitor cells than have other serotypes [196–198].

### Suicide genes and safety switches

While the motivation behind the incorporation of suicide genes and switches into CAR constructs was to mediate the severe adverse events commonly reported following extensive expansion of CAR T cells, they can also serve an alternative purpose. Using pharmacologic agents, the apoptotic pathway in CAR T cells can be activated, triggering selective cell death of the effector cells, without destroying bystander cells. Therefore, they can be valuable in the setting of T cell malignancies as they can prevent T cell aplasia. There are three main classes of suicide gene technologies, classified by the mechanism of action of the incorporated gene. They (i) convert non-toxic compounds to toxic drugs via metabolic pathways [199–202], (ii) induce dimerization of inducible caspase-9 [203, 204], or (iii) mediate ADCC using monoclonal antibodies [205–207]. Co-expression of the suicide gene with the CAR in a bicistronic vector would result in two populations of cells—those that express both the CAR and the suicide gene, and those that express neither. This strategy negates the risk of generating a CAR-positive population without the safety transgene; thus, enabling one to confidently eliminate the entire CAR-positive population and thereby, in the context of targeting T cell antigens, controlling T cell aplasia.

The first reported suicide gene utilized the herpes simplex virus thymidine kinase (HSV-TK) as a method of GvHD abrogation in the context of an allogeneic HSCT. Expression of HSV-TK in donor lymphocytes prior to their infusion into a HSCT patient allows for selective depletion of the donor lymphocytes in patients that developed signs of GvHD upon administration of ganciclovir [199–201]. Metabolism of ganciclovir by the thymidine kinase of HSV-TK results in a toxic substance, ultimately killing the cell [208]. However, there are a couple of limitations to this system including the potential for immunogenicity and the slow T cell depletion, which requires about 3 days [209–211].

More recently, the safety mechanism gaining the most attention has been the inclusion of an inducible caspase-9-based suicide gene (iCas9) into the CAR construct. Pharmacologic activation of the iCas9 results in effective and rapid elimination of CAR T cells. iCas9 inclusion in a CD19-CAR construct has been shown to regulate CAR T cells in a dose-dependent manner, allowing for either control over the CAR T cells to reduce toxicities, or complete elimination of all CAR T cells to facilitate B cell reconstitution [212, 213]. This is especially significant in cases with severe adverse events, such as GvHD or CRS. iCas9 has recently been included in CAR constructs containing an IL-15 gene to introduce control over CAR T cell function. The IL-15 gene arms the T cells to produce IL-15, which, while increasing T cell survival and enhancing specific cytotoxicity, can also result in unrestricted proliferation and increased toxicity. Inclusion of an iCas9 gene in these CAR constructs can provide control to this therapy and increase the safety profile [214]. In addition to CD19-CARs, iCas9 has been included in other CAR constructs including an anti-CD20-CAR, demonstrating enhanced tumor clearance in vivo and a 90% reduction in CAR T cells in the peripheral blood of mice following activation of the iCas9 suicide gene, compared to CAR T cells detected in peripheral blood of control mice [215]. Additionally, a GD2-CAR including the iCas9 gene is being assessed for the treatment of neuroblastoma (NCT01822652), sarcoma (NCT01953900), osteosarcoma, and melanoma (NCT02107963) in phase I clinical trials.

In terms of utilizing ADCC for CAR T cell clearance, administration of alemtuzumab, an anti-CD52 antibody commonly used in lymphodepleting regimens, has been tested in several studies. Specifically, alemtuzumab has been assessed for CD4-CAR T cell elimination following tumor cell eradication in NSG mice to prevent T cell aplasia [87]. Within 6 h following alemtuzumab infusion, > 95% of the CAR T cells had been depleted. This approach was also tested in two other preclinical CAR studies targeting AML, both showing excellent results [216, 217]. Multiple groups have also evaluated the retroviral transfer of human CD20 into T cells as a novel suicide gene mechanism for adoptive T cell therapy. Their data supports that infusion of the anti-CD20 antibody, rituximab, an approved antibody for in vivo therapeutic applications, results in efficient, specific elimination of CD20-positive T lymphocytes through ADCC [205, 206, 218]. Studies have also demonstrated that rituximab can eliminate CD20-positive cells in vivo through inducing complement-dependent cytotoxicity, a rapid and efficient mode of cell death [219]. CD20 co-expression with a CD123-CAR demonstrated strong and rapid anti-leukemia activity in a human AML mouse model. Upon the infusion of rituximab, CAR T cells were cleared and mice were successfully engrafted with human bone marrow cells, mimicking an allogeneic HSCT [217]. Thus, ADCC-based safety systems potentially allow for rapid and efficient elimination of CAR T cells [211].

An epitope-based marker/suicide gene system (RQR8) was recently developed to both track the transduced cells and selectively deplete them by combining epitopes from CD34 and CD20 [220]. Use of Miltenyi Biotec’s clinically approved CliniMACS CD34 system allows for selection of the CAR-modified T cells while the binding of rituximab results in ADCC and selective elimination of the adoptively transferred T cells. Co-expression of RQR8 with an anti-GD2 CAR demonstrated selection of CAR T cells with > 95% purity and clearance of > 97% of the CAR-positive population. This RQR8 system is currently being tested in clinical trials for the treatment of T cell non-Hodgkin lymphoma targeting TRBC1 (NCT03590574). Another polypeptide that has been designed to facilitate the selection of CAR-positive T cells, tracking of the cells in vivo and selective elimination as a safety mechanism, is the truncated human epidermal growth factor receptor (huEGFRt). Manipulation of this protein was done to remove intracellular signaling domains, leaving it with an intact epitope for binding cetuximab, an anti-EGFR monoclonal antibody. Modification of T cells with the CAR and huEGFRt allows for selection using GMP biotin immunomagnetic beads and biotinylated cetuximab, and tracking using flow cytometry or immunohistochemistry. Upon administration of cetuximab, CAR T cells become the targets for ADCC, resulting in in vivo depletion of CAR T cells. Successful T cell engraftment and ADCC-mediated CAR T cell elimination with cetuximab were demonstrated in a murine model [221]. The huEGFRt suicide mechanism is currently being assessed in a phase I clinical trial in an anti-MUC-16^ecto^ CAR construct to treat patients with recurrent MUC16^ecto+^ solid tumors (NCT02498912) [222].

A novel alternative approach to suicide genes is the generation of “ON-switch” CARs [223]. In this strategy, the CAR is a split receptor consisting of two distinct polypeptides: the antigen recognition domain and the intracellular signaling domain. In order to act as a functional receptor, the two peptides must first dimerize, achieved through activation by a dimerization-inducing small molecule. However, antigen stimulation is still required to facilitate a response. The small molecule can be titrated for optimal response, controlling the timing and dosage of active CAR T cells. Thus, removal of the small molecule can reversibly regulate CAR T cell activity. These ON-switch CAR T cells demonstrate specific cytotoxicity in vitro and in vivo only when exposed to the small molecule. In a mouse xenograft model, mice treated with ON-switch CAR T cells displayed a reduction in K562 cells engineered to express CD19, only in the presence of the small molecule, similar to mice treated with conventional CD19-CAR T cells. However, no benefit was seen in the absence of the small molecule. Given the tight control over CAR expression using this innovative approach, it has the potential to be adapted for T cell malignancies.

## Summary and conclusions

CAR therapies targeting CD19 have resulted in unparalleled success. However, there are many challenges in translating these therapies beyond the treatment of B cell malignancies. We have highlighted some of these challenges as it pertains to targeting T cell disease. While numerous antigens have been identified for the treatment of T cell malignancies, targeting of many of these antigens results in fratricide and T cell aplasia. Multiple gene editing approaches are being evaluated to prevent fratricide by reducing expression of the targeted antigen on CAR-modified cells. The identification of tumor-specific antigens would greatly enhance CAR therapy targeting T cell malignancies by avoiding fratricide. To date, only a few antigens with limited expression on normal T cells have been assessed as CAR targets to treat T cell malignancies; these include CD30, CD37, and CD1a. However, given their expression on only small subsets of T cell cancers, a focus on these antigens is unlikely to have a wide-ranging impact on the overall translation of CAR therapy for patients with T cell disease. In contrast, TRBC1 is expressed on a much larger population of T cells and therefore it is likely to be found on a comparatively higher percentage of T cell malignancies. To the best of our knowledge, only one study has evaluated anti-TRBC1-CAR T cell therapy. The data suggests that TRBC1 is a very promising marker for targeting T cell malignancies and the field would benefit from studies further developing this therapy.

Among other target antigens, CD5 has emerged as a promising candidate given its ability to rapidly downregulate from the cell surface upon interaction with the CD5-CAR. Therefore, only transient and limited fratricide is observed, allowing for successful expansion of CD5-CAR T cells. While targeting CD5 or other T cell antigens using gene-edited CAR T cells may overcome the issue of fratricide, the concern regarding T cell aplasia has not been addressed. The potential for life-threatening T cell aplasia emphasizes the need for a safety mechanism that is completely effective at eliminating CAR T cells following tumor eradication. Safer alternatives other than bridging to an allogeneic HSCT must be explored to limit CAR T cell persistence. Adjusting the effector cell type to NK cells, NK-92 cells, or γδ T cells can limit the risk of a memory cell immune response against a T cell antigen. However, given that NK-92 cells require irradiation prior to infusion in a patient, their therapeutic effect may be limited. mRNA electroporation or AAV delivery systems, which result in transient CAR expression, could be utilized, thereby allowing for restoration of normal T cell immunity once the CAR effect has diminished. Additionally, the use of iCas9 and ADCC-based suicide genes, as well as other CAR safety switches should be explored in the context of T cell malignancies.

However, these strategies do not address the pressing issue of isolating normal healthy T cells from malignant T cells upon leukapheresis, prior to modification with a CAR construct. A perfect system needs to be in place to prevent transduction of a leukemic blast, a phenomenon that has occurred in a B-ALL patient, resulting in relapse and ultimately death. In order to eliminate any risk of this event, third party donor cells must be used. Disruption of TCR expression through genome editing of the TRAC locus is required to prevent GvHD, when using allogeneic αβ T cells for CAR expression. However, NK cells and γδ T cells can both be used in an allogeneic setting given their MHC-independent activation, and are thus unlikely to cause GvHD. Use of allogeneic CAR-modified cells also addresses the challenges of high cost and difficulty of production, since healthy donor cells can be expanded more easily and cryopreserved as an off-the-shelf therapy until they are required for use. Additionally, allogeneic cell delivery allows for titratable dosing as well as multiple infusions, if such is required.

**Fig. 2:**
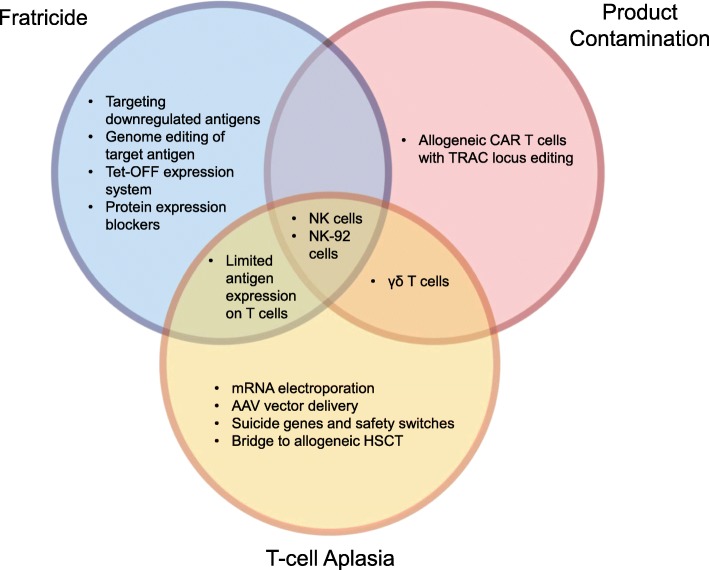
Venn diagram representing challenges and solutions in targeting T cell antigens with CAR therapy. Each circle represents a hurdle associated with translation of CAR therapy to T cell disease—fratricide, T cell aplasia, and product contamination. As seen in the figure, only the use of NK cells or NK-92 cells as the CAR-effector cell can potentially address all three issues concurrently. However, using NK cells or NK-92 cells comes with its own limitations as previously described. All other approaches require multiple modifications to generate a translatable CAR product to target T cell disease. Potential alternative solutions such as use of γδ T cells as the CAR-effector cell, transient CAR expression with mRNA electroporation or AAV viral delivery, as well as incorporating suicide genes and safety switches, remain largely unexplored. A greater focus on implementing such strategies is required to enable successful translation of this therapy for T cell malignancies

Many avenues are currently being explored to enhance the safety and efficacy of CAR therapy. However, the majority of these strategies do not address all three main challenges to utilizing CAR therapy to treat T cell malignancies. Of the approaches evaluated in this review, only those incorporating NK cells or NK-92 cells can potentially overcome all of these primary challenges (Fig. 2). NK cells (i) are non-alloreactive and can be obtained from healthy donors, eliminating risk of product contamination; (ii) do not form memory responses, preventing T cell aplasia; and (iii) do not express the same antigen repertoire as T cells, avoiding fratricidal concerns. CD7 is an exception as it is expressed on NK cells and therefore fratricide could occur. While several groups have published studies with CAR NK-92 cells targeting T cell malignancies, more effort needs to be put into using primary NK cells for targeting this disease, especially given the limitations of NK-92 cells. Other, equally promising approaches, such as utilizing γδ T cells as the cellular vehicle for CAR therapy represents an alternative, less studied approach. Similar to NK cells, γδ T cells are non-alloreactive and are unlikely to form a memory response against a T cell antigen. γδ T cells are likely to succumb to fratricide in certain circumstances; however, targeting an antigen such as CD5 that results in only transient and limited fratricide may be especially advantageous. Furthermore, γδ T cells exhibit innate MHC-independent mechanisms of cytotoxicity by which they can recognize tumor cells. Thus, CAR therapy using γδ T cells represents an understudied avenue with the potential of developing into a superior cellular product.

Many advances have been made toward translating CAR therapy for the treatment of T cell malignancies. Both academia and industry are focused on the identification of tumor-specific antigens to enhance the safety and efficacy of CAR T cell products as well as on the development of superior cellular products. Unfortunately, due to vast variability in the design and execution of preclinical studies, it is often difficult to compare the different strategies. However, the numerous preclinical and clinical studies currently underway provide optimism for successful translation of this therapy to treat this aggressive and challenging group of diseases.

## Data Availability

Not applicable.

## Abbreviations

AAV: Adeno-associated virus
ADCC: Antibody-dependent cellular cytotoxicity
AITL: Angioimmunoblastic T cell lymphoma
ALCL: Anaplastic large cell lymphoma
AML: Acute myeloid leukemia
APC: Antigen presenting cell
ATLL: Adult T cell leukemia/lymphoma
B-ALL: B cell acute lymphoblastic leukemia
BCMA: B cell maturation antigen
CAR: Chimeric antigen receptor
cGMP: Current good manufacturing process
CRS: Cytokine release syndrome
CTCL: Cutaneous T cell lymphoma
DLBCL: Diffuse large B cell lymphoma
EATL: Enteropathy-associated T cell lymphoma
ENKTL: Extranodal natural killer T cell lymphoma
epCAM: Epithelial cell adhesion molecule
ETP-ALL: Early T cell precursor acute lymphoblastic leukemia
FDA: Food and Drug Administration
FPPS: Farnesyl pyrophosphate synthase
GvHD: Graft versus host disease
HER2: Human epidermal growth factor receptor
HLA: Human leukocyte antigen
HSCT: Hematopoietic stem cell transplantation
HSTCL: Hepatosplenic T cell lymphoma
HSV-TK: Herpes simplex virus thymidine kinase
HTLV1: Human T cell lymphocytic virus type 1
huEGFRt: Truncated human epidermal growth factor receptor
ICAM1: Intercellular adhesion molecule 1
iCas9: Inducible Cas9
IL-2: Interleukin-2
IPP: Isopentenyl pyrophosphate
KIR: Killer-cell immunoglobulin-like receptor
MCSP: Melanoma-associated chondroitin sulfate proteoglycan
MF: Mycosis fungoides
MHC: Major histocompatibility complex
MICA/B: MHC class I chain-related protein A/B
NK: Natural killer
NKG2D: NK group 2 member D receptor
NOS: Not otherwise specified
NSG: NOD scid IL2Rγ-chain
PD-1: Programmed cell death receptor 1
PDX: Patient-derived xenograft
PEBL: Protein expression blocker
PTCL: Peripheral T cell lymphoma
scFv: Single-chain variable fragment
SS: Sezary syndrome
TALEN: Transcription activator-like effector nuclease
T-ALL: T cell acute lymphoblastic leukemia
TCR: T cell receptor
TIL: Tumor-infiltrating lymphocyte
T-LGL: T cell large granular lymphocytic leukemia
T-LLy: T-lymphoblastic lymphoma
TNF: Tumor necrosis factor
TNFR: Tumor necrosis factor receptor
T-PLL: T-prolymphocytic leukemia
TRAC: T cell receptor α constant
TRAF: TNF receptor-associated factor
TRAIL: TNF-related apoptosis-inducing ligand
TRBC1: T cell receptor beta constant 1
ULBPs: UL16 binding proteins
VIP: Vasoactive intestinal peptide
γδ T cell: Gamma delta T cell
